# Construction and Characterization of Long Non-Coding RNA-Associated Networks to Reveal Potential Prognostic Biomarkers in Human Lung Adenocarcinoma

**DOI:** 10.3389/fonc.2021.720400

**Published:** 2021-08-27

**Authors:** Wenting Zhou, Chen Bai, Chaojun Long, Li Hu, Yanfei Zheng

**Affiliations:** School of Traditional Chinese Medicine, Beijing University of Chinese Medicine, Beijing, China

**Keywords:** lung adenocarcinoma, lncRNA, network analysis, prognosis biomarkers, m6A

## Abstract

Lung adenocarcinoma (LUAD) is one type of the malignant tumors with high morbidity and mortality. The molecular mechanism of LUAD is still unclear. Studies demonstrate that lncRNAs play crucial roles in LUAD tumorigenesis and can be used as prognosis biomarkers. Thus, in this study, to identify more robust biomarkers of LUAD, we firstly constructed LUAD-related lncRNA-TF network and performed topological analyses for the network. Results showed that the network was a scale-free network, and some hub genes with high clinical values were identified, such as lncRNA RP11-173A16 and TF ZBTB37. Module analysis on the network revealed one close lncRNA module, which had good prognosis performance in LUAD. Furthermore, through integrating ceRNAs strategy and TF regulatory information, we identified some lncRNA-TF positive feedback loops. Prognostic analysis revealed that ELK4- and BDP1-related feedback loops were significant. Secondly, we constructed the lncRNA-m6A regulator network by merging all the high correlated lncRNA-m6A regulator pairs. Based on the network analysis results, some key m6A-related lncRNAs were identified, such as MIR497HG, FENDRR, and RP1-199J3. We also investigated the relationships between these lncRNAs and immune cell infiltration. Results showed that these m6A-related lncRNAs were high correlated with tumor immunity. All these results provide a new perspective for the diagnostic biomarker and therapeutic target identification of LUAD.

## Introduction

Lung adenocarcinoma (LUAD) is one of the key subtypes of lung cancer with high morbidity and mortality in the world (1, 2). LUAD is also the major cause of cancer-related deaths and is hard to diagnose. Nearly 15% of patients with LUAD sustained life for no more than 5 years. Despite the great efforts in cancer diagnostic and therapeutic strategies that have been made, such as PD-L1 immunotherapy, the clinical outcomes of LUAD have not substantially improved, which are induced by overdue diagnosis and tumor metastasis. Thus, it is urgent to identify the molecular mechanism to unveil the initiation and progression of LUAD. Furthermore, identification of molecular diagnosis and prognosis biomarkers from omics data is also important.

The long non-coding RNA (lncRNA) is a novel type of transcript with length more than 200 nucleotides (3, 4). Previous studies found that lncRNAs are implicated in numerous biological processes in multiple diseases, such as cancer and cardiovascular diseases (5–7). Clinically, dysregulated lncRNAs may act as diagnosis and prognosis biomarkers and therapeutic targets for cancers (8, 9). The molecular mechanisms of lncRNAs in diseases are various, such as formatting competing endogenous RNAs (ceRNAs), histone modification, and binding proteins (10, 11). In the field of LUAD, some studies have identified that lncRNAs are the crucial regulators in tumor progression. For instance, Cong et al. found that long non-coding RNA LINC00665 could function as a ceRNA to regulate AKR1B10-ERK signaling by sponging miR-98 to promote lung adenocarcinoma progression (12). Wang et al. performed a bioinformatics analysis by integrating TCGA LUAD data and revealed that lncRNA CTB-193M12.5 was a prognostic factor in lung adenocarcinoma (13). Peng et al. found a robust prognostic signature of two lncRNAs (C1orf132 and TMPO-AS1) for stage I-II LUAD patients without adjunctive therapy (14). In addition, Huang et al. found that lncRNA LINC00520 could interact with miR-3611 and target to FOXP3 to facilitate cell proliferative and migratory abilities in LUAD, while FOXP3 was also the upstream transcription factor (TF) of LINC00520. This positive feedback containing FOXP3, miR-3611, and LINC00520 provides a potential novel insight for treatment of LUAD (preprint). Furthermore, methylation of N6 adenosine (m6A) regulation is a novel proposed regulatory mechanism, which is also the most widely distributed methylation modification in eukaryotic RNAs (15, 16). Studies demonstrated that m6A methylation of lncRNAs determined the clinical outcome in LUAD. For instance, Yu et al. found that ALKBH5-mediated m6A demethylation of lncRNA RMRP plays an oncogenic role in lung adenocarcinoma. ALKBH5 manipulation mediated RMRP suppression, which may be considered as a promising therapeutic target for lung adenocarcinoma (17). Xu et al. found a risk model with 12 m6A-related lncRNAs that could be used as an independent predictor of prognoses in LUAD (18). These studies inspired us to comprehensively investigate the lncRNA-related gene crosstalks to identify more diagnosis or prognosis biomarkers and identify more novel molecular therapeutic targets in LUAD.

In this study, firstly, we proposed a novel pipeline to construct LUAD-related lncRNA-TF network and performed topological analysis on the network. Results showed that the network was a scale-free network, and some hub genes were identified. Then we performed module analysis in the network. One lncRNA-related function module was identified by MCODE. This functional module showed good prognosis performances in LUAD. Furthermore, through integrating ceRNAs strategy and TF regulatory information, we identified some lncRNA-TF positive feedback loops, which also exhibited high prognosis performances. Secondly, we constructed the lncRNA-m6A regulator network by collecting all the high correlated lncRNA-m6A regulator pairs. Based on the network analysis results, some key m6A-regulated lncRNAs were identified. We also investigated the relationships between these lncRNAs and immune cell infiltration. These results shed new light into the diagnostic biomarker and therapeutic target identification of LUAD.

## Materials and Methods

### Lung Adenocarcinoma-Related Expression Data and Genetic Alterations Data

TCGA lung adenocarcinoma (LUAD)-related gene expression data and follow-up clinical data were obtained from XENA (https://xenabrowser.net/hub/). All the raw data are provided in **Table S1**. Genes with 0 expression value in more than 70% of samples were removed. As a result, processed LUAD gene expression data and matched clinical data contain 585 samples. TCGA LUAD genetic alteration data were obtained from cBioportal for Cancer Genomics. The list of m6A regulators and TFs was obtained from other studies. Gene expression comparison analysis was performed by GEPIA (19). Independent validation cohorts were downloaded from GEO database with the accession numbers of GSE30219 and GSE3141.

### Obtain ceRNA Interactions

For TF-mRNA interactions, 423,975 miRNA-mRNA interactions of starBase (including 386 miRNAs and 13,861 mRNAs) were used to identify TF-miRNA interactions by mapping the TFs into the miRNA-mRNA interactions (20). TF names were downloaded from AnimalTFDB. For lncRNA-miRNA interactions, we downloaded all lncRNA sequences from GENCODE database and the miRNAs sequences from miRbase database. Miranda algorithm (default parameters: Score S >= 140 and Energy E <= 7.0) was then used to call significant miRNA-lncRNA interactions (21).

### Construction of LUAD-Related lncRNA-TF Network and Network Analysis

Previous studies have demonstrated that lncRNAs can regulate mRNA expression *via* targeting common miRNAs. Hypergeometric test is usually used as an effective tool to identify significant lncRNA-mRNA pairs through calculation of the number of common miRNAs. Thus, we firstly performed the hypergeometric test to call significant lncRNA-TF pairs based on the above miRNA-TF and miRNA-lncRNA interactions. The hypergeometric test formula was

$$p-value=1-\sum_{i=0}^{r-1}\frac{\left(\begin{array}{c} t\\ i \end{array}\right)\left(\begin{array}{c} m-t\\ n-i \end{array}\right)}{\left(\begin{array}{c} m\\ n \end{array}\right)}$$

where, *m* is the number of miRNAs in this study, *n* is the number of miRNAs that interact with a lncRNA, *t* is the number of miRNAs that interact with a TF, and *r* is the number of both-targeted miRNAs between TF and lncRNA.

Secondly, we calculated Pearson correlation coefficients (PCC) for all lncRNA-TF pairs. LncRNA-TF pairs with PCC>0.6 and P-value<0.01 were considered as significant lncRNA-TF ceRNA pairs. Here, we also performed weighted gene co-expression network analysis (WGCNA) to identify lncRNA-TF pairs and compared the results between PCC and WGCNA. The threshold of lncRNA-TF pairs was weight >0.5. Additionally, TF-mapped PPI interactions were also extracted from HPRD database. The LUAD-related lncRNA-TF network was constructed by merging all significant lncRNA-TF ceRNA pairs and TF-TF PPI pairs. Cytoscape V3.6.0 was used for network visualization. R package of “igraph” was used to perform network topological analyses, such as network degree, cluster coefficient, and average short path length. In this study, hub genes were defined as the top 10% nodes (containing both lncRNAs and TFs) with the highest degrees in the LUAD-related lncRNA-TF network.

Furthermore, we imported the LUAD-related lncRNA-TF network to the Cytoscape software and used the “MCODE” plug-in to locate functional lncRNA-TF modules (default parameters).

### Identify lncRNA-TF Positive Feedback Loops

LncRNAs and TFs can competitively target miRNAs and form ceRNA pairs. Meanwhile, TFs are important transcriptional regulators and exert regulatory function by binding to promoter or enhancer regions of DNA. In the previous step, we have identified lncRNA-TF ceRNA pairs. Then we devoted to identify transregulatory pairs between lncRNAs and TFs. We downloaded the permissive enhancers and lncRNA genomic annotation data from FANTOM5 and GENCODE. Secondly, using FIMO software, we identified TF binding sites in both DNA elements (22). LncRNA promoters were defined as the +/−2,000 bps from TSS. LncRNA enhancers were defined as the elements that were located in more than +/−2,000 bps of the lncRNA TSS. For the motif analysis, we performed FIMO with a P-value <1e–4 to scan promoter and enhancer regions (23). LncRNA-TF positive feedback loops were identified by integrating TF motif binding and ceRNA relationships.

### Construction of LUAD-Related lncRNA-m6A Regulators Network

We downloaded the m6A regulator list from the previous study (24). Previous studies have demonstrated that m6A regulators could bind lncRNAs and trigger their expression (25, 26). Thus, we calculated the Pearson correlation coefficients (PCC) for all lncRNAs and m6A regulators. LncRNA-m6A regulator pairs with PCC>0.6 were reserved, and the network was constructed by merging all lncRNA-m6A regulator pairs.

### Survival Analysis

Hazards Ratio (HR) analysis of TFs and lncRNAs in LUAD was performed by GEPIA2 by using Mantel–Cox test. For single gene–based survival analysis, patients were classified into high-Exp group and low-Exp group based on mean expression. To evaluate the prognostic effect of multiple genes, a risk score model was used to implement survival analysis as follows:

$$\text{RiskScore}=\sum_{\text{i}=1}^{\text{n}}{\text{r}}_{\text{i}}\text{Exp}(\text{i})$$

Where, *r_i_* represents the Univariate Cox regression coefficient of gene i from gene set, *Exp (i)* represents the expression value of gene i in corresponding patient, and *n* represents the number of genes in gene set. The mean risk score was used to classify patients into high-risk and low-risk groups. A Kaplan-Meier survival curve was performed for different patient groups. Log-rank test (P < 0.05) was used to yield statistical significance.

### Immune Cell Infiltration of lncRNAs in LUAD Patients

Infiltration estimation for all LUAD patients was downloaded from TIMER2 database (27). The potential role of lncRNAs in cell infiltration was estimated by calculating the correlation between lncRNA expression and infiltration estimation scores.

## Results

### Construction and Analysis of LUAD-Related lncRNA-TF Network

**Figure 1:**
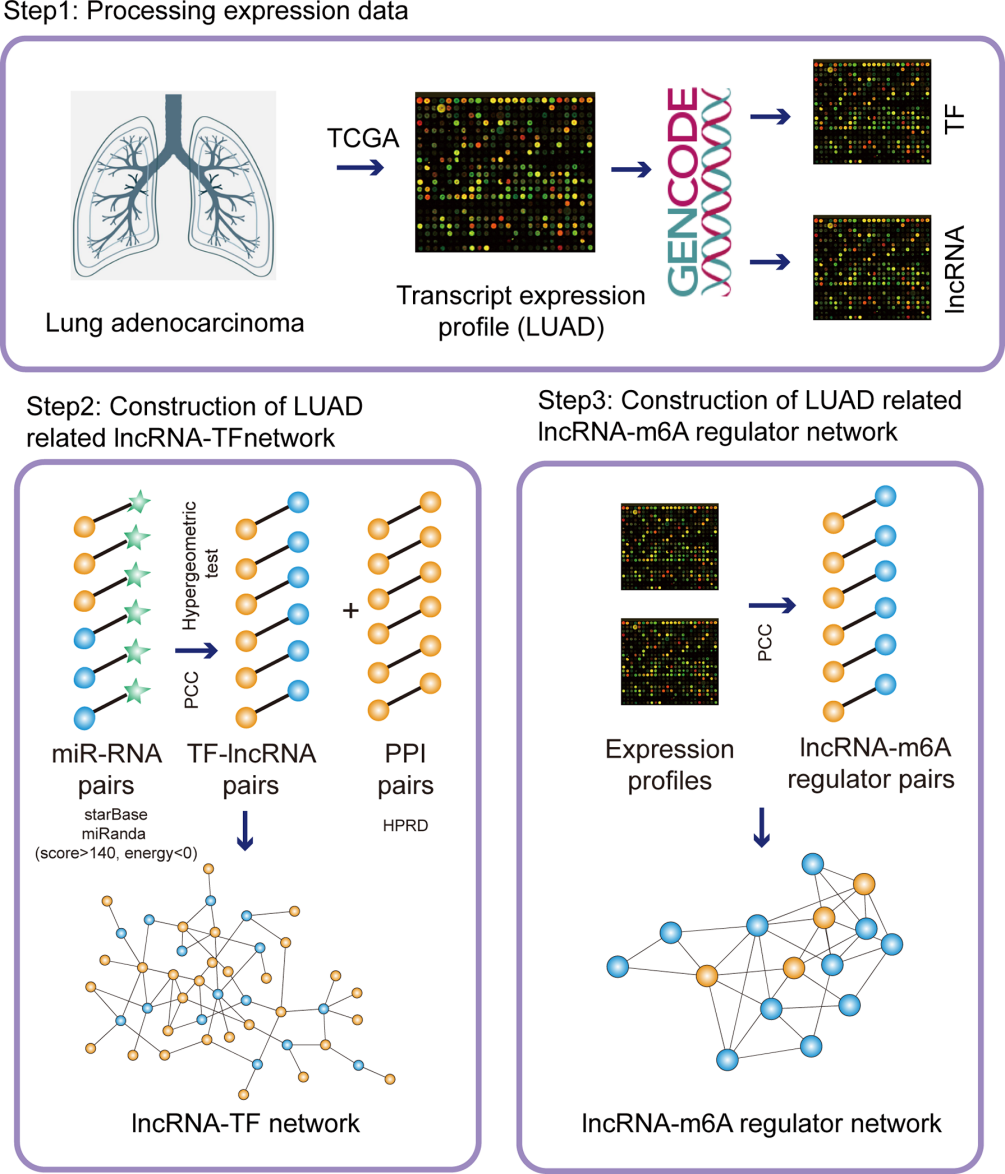
The pipelines of network construction. Firstly, we downloaded the transcripts expression from TCGA database and separated the lncRNA/TF expression. Secondly, we integrated miRNA-RNA interactions, co-expressed lncRNA-TF pairs, and PPI network to construct LUAD-related lncRNA-TF network. Thirdly, we integrated co-expressed lncRNA-m6A regulator pairs to construct LUAD-related lncRNA-m6A regulator network.

**Figure 2:**
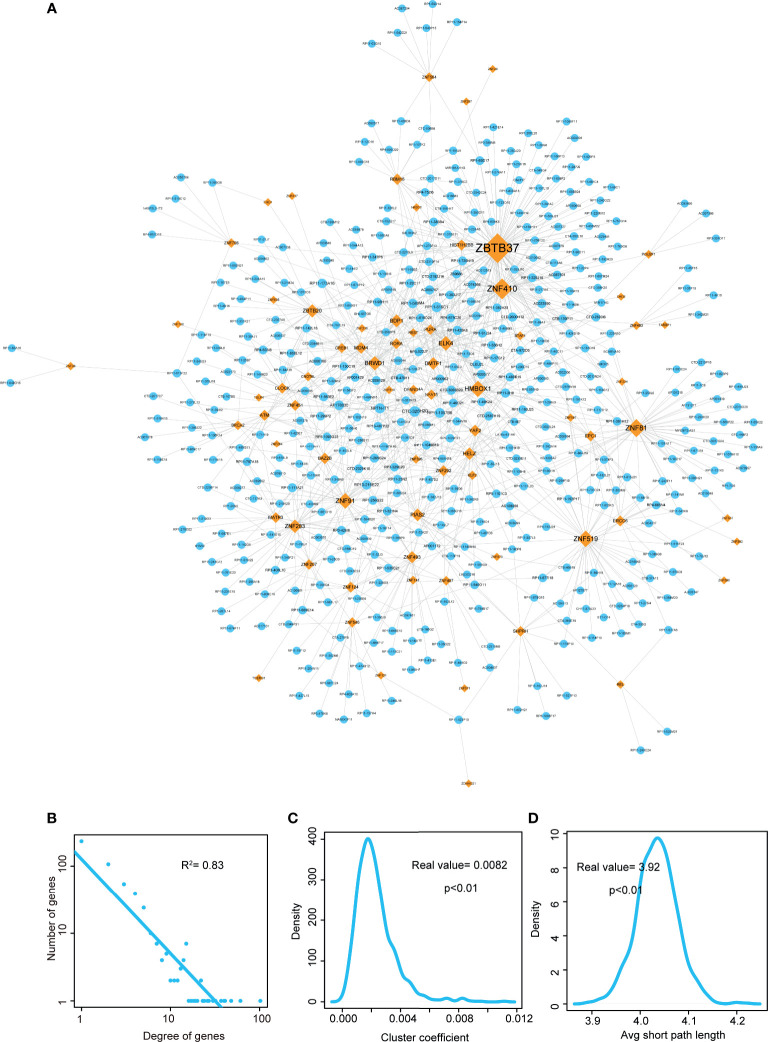
Visualization and topological features of LUAD-related lncRNA-TF network. **(A)** Cytoscape network visualization of LUAD-related lncRNA-TF network. LncRNA nodes are represented in orange, and TF nodes are represented in blue. Network node degrees are represented by node size. **(B)** Degree distributions of the LUAD-related lncRNA-TF network. **(C)** Cluster coefficient distributions of LUAD-related lncRNA-TF network and 1,000 times permutation networks. Cluster coefficient of LUAD-related lncRNA-TF network was larger than in permutation cases (p-value < 0.01). **(D)** Average short path lengths of LUAD-related lncRNA-TF network and 1,000 times permutation networks. Average short path length in real network was smaller than in permutation cases (p-value < 0.01).

LncRNAs have been considered as the crucial regulators in the procession of tumorigenesis. TF-lncRNA crosstalks are also important components in lncRNA-related functional mechanisms. Here, we firstly focused on identifying potential lncRNA regulators in LUAD based on lncRNA-TF network. To do it, we downloaded the gene expression profiles from TCGA portal and processed the expression data. Additionally, we downloaded the miRNA-mRNA crosstalks from starBase and identified miRNA-lncRNA crosstalks *via* miRanda tools. Based on ceRNA theory, we proposed a protocol by integrating hypergeometric test and Pearson correlation coefficients to construct LUAD-related lncRNA-TF network (**Figure 1**). As a result, the LUAD-related lncRNA-TF network constituted 78 TF nodes, 446 lncRNA nodes, and 982 crosstalks (**Figure 2A**).

Then we performed topological analyses on the LUAD-related lncRNA-TF network and identified the topologically crucial lncRNA crosstalks. Firstly, we performed degree distribution on the network and found all nodes followed power law distribution (**Figure 2B**, R^2^ = 0.83), suggesting that the LUAD-related lncRNA-TF network had scale-free characteristic. This result also suggested that in the network, a small group of genes with high degree (considered as hub genes) linked to the other most genes. Secondly, cluster coefficient of the real network and cluster coefficients of 1,000 times permutation networks were calculated. As a result, cluster coefficient of LUAD-related lncRNA-TF network was significantly larger than that of 1,000 times permutation networks (**Figure 2C**, p<0.01), indicating that the network had strong aggregation capability. Thirdly, we calculated average short path lengths for the real network and 1,000 times permutation networks. Results showed that short path length of the real network was significantly smaller than short path lengths of 1,000 times permutation networks (**Figure 2D**, p<0.01), suggesting that the lncRNA-TF network had reduced global efficiency. All these results implied that the LUAD-related lncRNA-TF network could be used to identify crucial regulators of LUAD.

In addition, we also integrated WGCNA method and hypergeometric test to identify lncRNA-TF crosstalks. As a result, 69 lncRNAs, 12 TFs, and 97 edges were consisted of the WGCNA-based lncRNA-TF network (**Figure S1**). In the network, some hub TFs of LUAD-related lncRNA-TF network were also identified as central regulators, such as ZNF410, ZBTB20, and MATR3. However, a TF hub named as YAF2 was considered as the novel core regulator in the WGCNA network. This result indicated that the new integrative method could help reveal more knowledge in tumorigenesis of LUAD.

### Identification of Hub Genes in LUAD-Related lncRNA-TF Network

**Figure 3:**
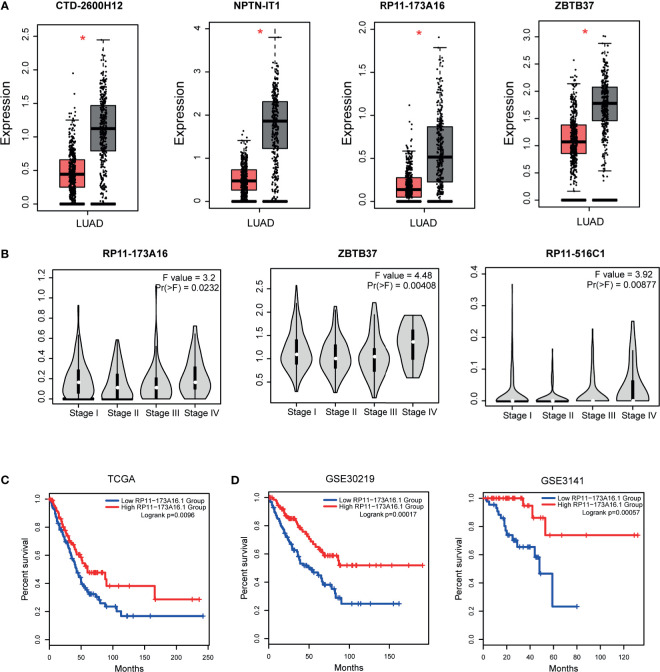
Expression and clinical value of hub genes. **(A)** The expression patterns of one TF hubs (ZBTB37) and three lncRNA hubs (CTD-2600H12, NPTN-IT1, and RP11-173A16) with the highest degrees in TCGA LUAD cohorts. **(B)** Three hub genes (RP11-173A16, RP11-516C1, and ZBTB37) were selected to detect their expression in different stages of lung adenocarcinoma. **(C)** The prognostic effect of hub lncRNA RP11-173A16 in TCGA cohorts. **(D)** The prognostic effects of hub lncRNA RP11-173A16 in other two independent validation cohorts. *represents p < 0.05, tumor vs control group http://pathwax.sbc.su.se.

Previous studies have demonstrated that topologically crucial genes in biological networks maintained crucial functions in physiological and pathological processes. Thus, we then identified the topologically crucial genes by calculating the network degree for each node. We extracted the top 10% nodes with the highest degrees from the LUAD-related lncRNA-TF network and defined these genes as hub genes. As a result, 31 lncRNAs and 22 TFs were extracted as hub genes. We detected the expression of these hub genes in LUAD dataset, and results showed that hub lncRNAs and hub TFs showed significant expression changes in tumor and control samples (**Figures 3A** and **S2**). This result also implied that the topologically crucial regulators in the network participated in the regulatory processes of cancers. Furthermore, we also investigated the role of hub genes in classifying cancer subtypes. Results showed that hub genes had potential in malignant lesion staging (**Figure 3B**). Interestingly, we found that hub lncRNAs had the potential to be used as prognosis markers. For example, the high expression of hub lncRNA RP11-173A16 showed a good prognosis in TCGA cohorts (**Figure 3C**). Moreover, it was also considered as a robust prognosis marker in independent validation datasets (**Figure 3D**). Furthermore, we also used OncoVAR database for analysis of the oncogenic driver genes in LUAD. Results showed that hub TF ATM was a driver gene in LUAD (**Table S2**). And we also listed the ATM-associated mutations in LUAD (**Figure S3**) (28).

### Identification of Functional lncRNA-TF Modules

**Figure 4:**
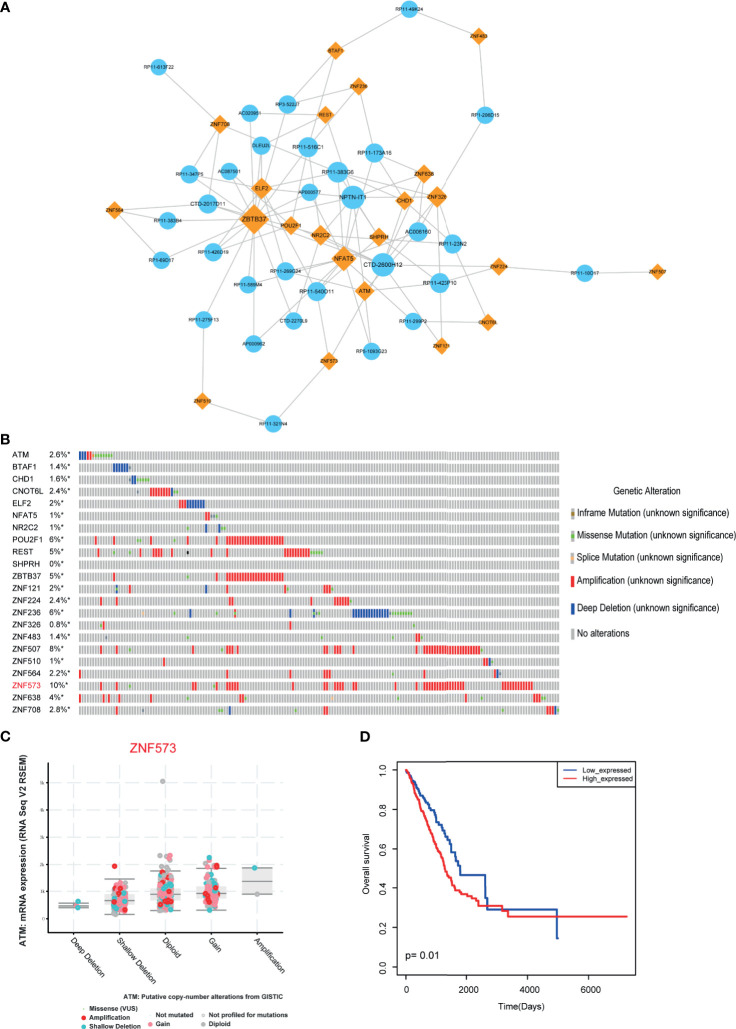
Module analysis of LUAD-related lncRNA-TF network. **(A)** Cytoscape visualization of module in LUAD-related lncRNA-TF network. **(B)** Genetic alteration information of TFs in modules. **(C)** Genetic alteration has impact on TF expression. **(D)** The prognosis effects of the lncRNA-related module. *represents genes were selected in the dataset.

Biological networks are often too large to interpret their certain biological functions. However, functional modules of the network may be more useful for uncovering certain regulatory mechanisms. Functional modules have also been widely used to explore the mechanism involved in many aspects, such as cancers and cardiovascular diseases (29, 30). Thus, in this study, we used “MCODE” software to locate the functional models in LUAD-related lncRNA-TF network. As a result, one functional module was identified with default parameters (**Figure 4A**). This module constituted 22 TFs, 31 lncRNAs, and 100 edges. Some TFs in the module have been demonstrated to involve in cancer procession, such as CHD1, NFAT5, and POU2F1. Then we focused on investigating the variations of these TFs. We called the genetic alteration information of these genes from TCGA portal. Results showed that these TFs had amplification alterations in LUAD (**Figure 4B**), indicating these TFs showing high expression patterns in cancer were regulated by genetic alterations. ZNF573 was the high-mutated TF with 10% alteration frequency. RNA-seq results also validated that the alteration of genes had an impact on gene expression (**Figure 4C**). Furthermore, we also used risk score model to detect the prognosis effect of the modules in LUAD cohorts. Results showed that this functional module had prognosis potential (**Figure 4D**).

### Identification of lncRNA-TF Positive Feedback Loops

**Figure 5:**
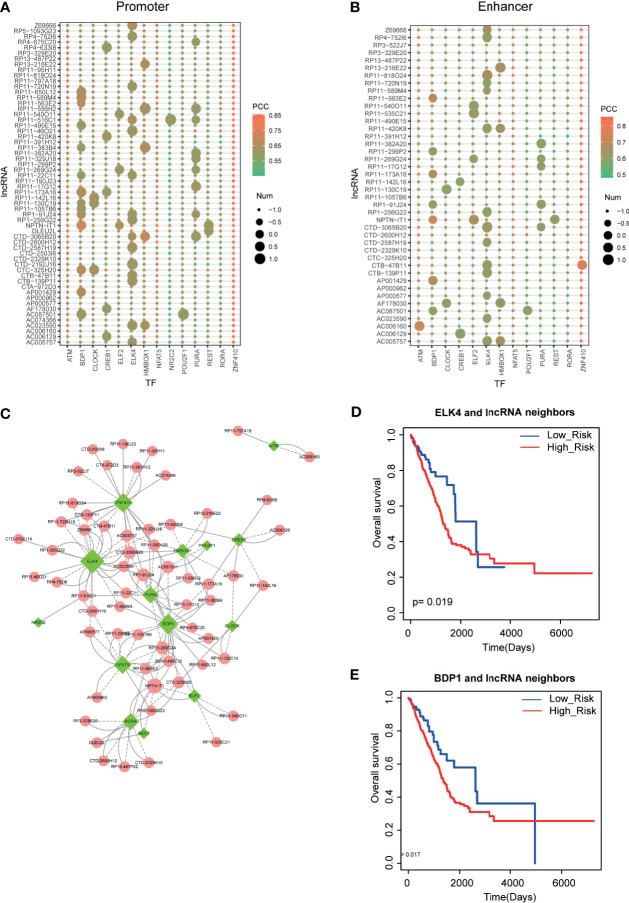
Identification of lncRNA-TF positive feedback loops. **(A, B)** Motif scanning results of lncRNA promoter and enhancer regions. Heatmap represents the Pearson correlation coefficients between lncRNAs and TFs. Size represents the number of motifs in DNA regions. **(C)** Cytoscape visualization of lncRNA-TF positive feedback loops. **(D, E)** The prognostic effects of ELK4-lncRNA feedback pairs and BDP1-lncRNA feedback pairs in LUAD.

Recent studies have demonstrated that TFs and lncRNAs could form positive feedback loops to exert biological functions. Thus, to investigate the LUAD-related lncRNA-TF feedback loops, we collected the regulatory elements of all lncRNAs from GENCODE and FANTOM5. Then we used motif scanning software to calculate binding capacities between TFs and lncRNAs. As a result, we found TF binding sites in the promoters and enhancers of lncRNAs, respectively (**Figures 5A, B** **)**. In detail, we found that some TFs, such as BDP1 and ELK4, had a large number of TF binding sites in these lncRNA upstream DNA elements. Additionally, some TFs, such as ZNF410 and NFAT5, were highly co-expressed with lncRNAs but had non-motif binding affinity characteristic, implying that these TFs might regulate lncRNA expression in co-factor. All these results implied the broad functions of these TFs in LUAD. Then we merged all the lncRNA-TF positive feedback loops into the network (**Figure 5C**). ELK4, BDP1, and ZNF410 were the hub TFs in feedback loop networks. More importantly, we also detected the clinical value of these lncRNA-TF feedback loops. Results showed that these lncRNA-TF feedback loops had good prognostic effects (**Figures 5D, E** **)**.

### Construction and Analysis of lncRNA-m6A Regulator Network

**Figure 6:**
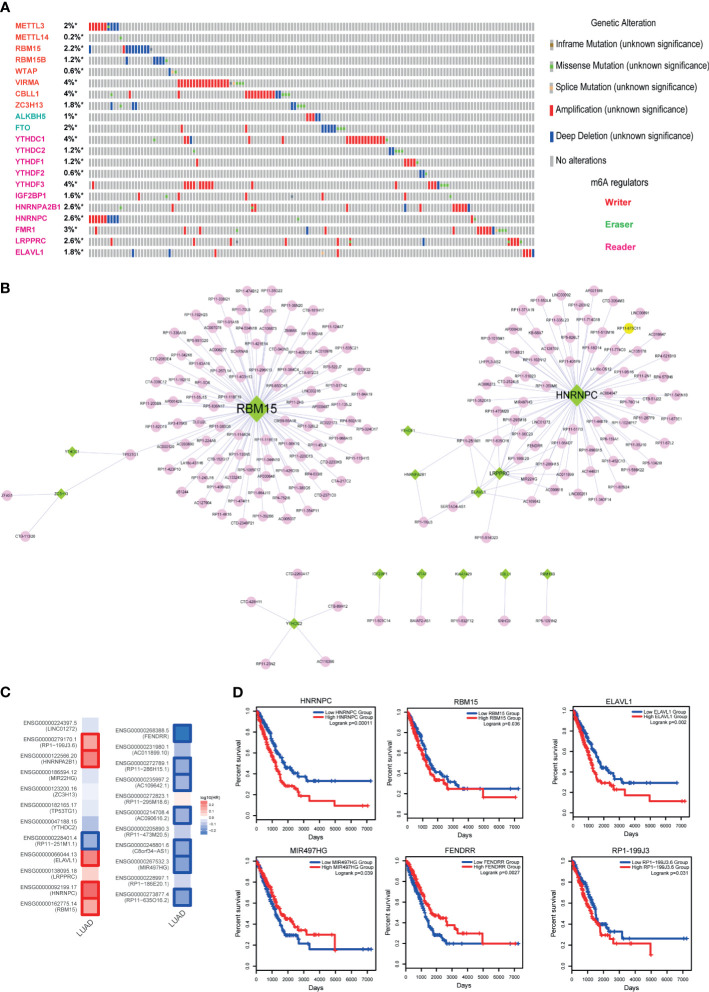
Identification of m6A regulator-lncRNA pairs in LUAD. **(A)** Genetic alteration information of m6A regulators in LUAD. **(B)** High-correlated m6A regulator-lncRNA pairs. Green nodes represent m6A regulators, and pink nodes represent lncRNAs. **(C)** Hazard ratios of 23 key genes of the lncRNA-m6A regulator network. **(D)** The Kaplan-Meier survival curves of key lncRNAs and m6A regulators. *represents genes were selected in the dataset.

Methylation of N6 adenosine (m6A) regulation is considered as a crucial regulatory mechanism in tumorigenesis. Here, to investigate the role of m6A regulators in LUAD, we firstly called the genetic alterations of 21 m6A regulators in LUAD. Results showed that these regulators were mutated in LUAD, although the alteration frequencies were not high (**Figure 6A**, less than 4%). M6A modifications of lncRNAs were also demonstrated as the powerful evidences in cancer researches (31, 32). Studies also found that m6A regulators could trigger lncRNA expression. Thus, this motivated us to construct the lncRNA-m6A regulator crosstalks *via* calculating co-expression coefficients. As a result, we calculated the Pearson correlation coefficients between all lncRNAs and 21 m6A regulators and extracted the lncRNA-m6A regulator pairs with PCC>0.6 or PCC<−0.6 into the crosstalk network. As a result, this network consisted of 15 m6A regulators, 178 lncRNAs, and 200 edges (**Figure 6B**). Firstly, we calculated the degree for the network, and we could see that RBM15 and HNRNPC were the topologically central nodes, implying these two m6A regulators might participate in more biological processes in LUAD. Here, we only focused on the genes with high degree (degree >2) in the network because of the specific network characteristic. As a result, 23 genes were extracted as the key genes, which included 7 m6A regulators and 16 lncRNAs. To investigate the prognostic effects of these genes, we calculated the Hazard ratios in TCGA LUAD datasets *via* GEPIA2. Results showed that some lncRNAs and m6A regulators were high related to the LUAD survival, such as RBM15, FENDRR, and HNRNPC (**Figure 6C**). Then we used log-rank test to investigate the prognostic effects of these genes. Results also validated that m6A regulators and m6A-related lncRNAs had strong prognostic effects, indicating that m6A modification could be used as a robust biomarker in LUAD (**Figures 6C, D**).

### Immune Cell Infiltration of m6A-Related lncRNAs in LUAD

**Figure 7:**
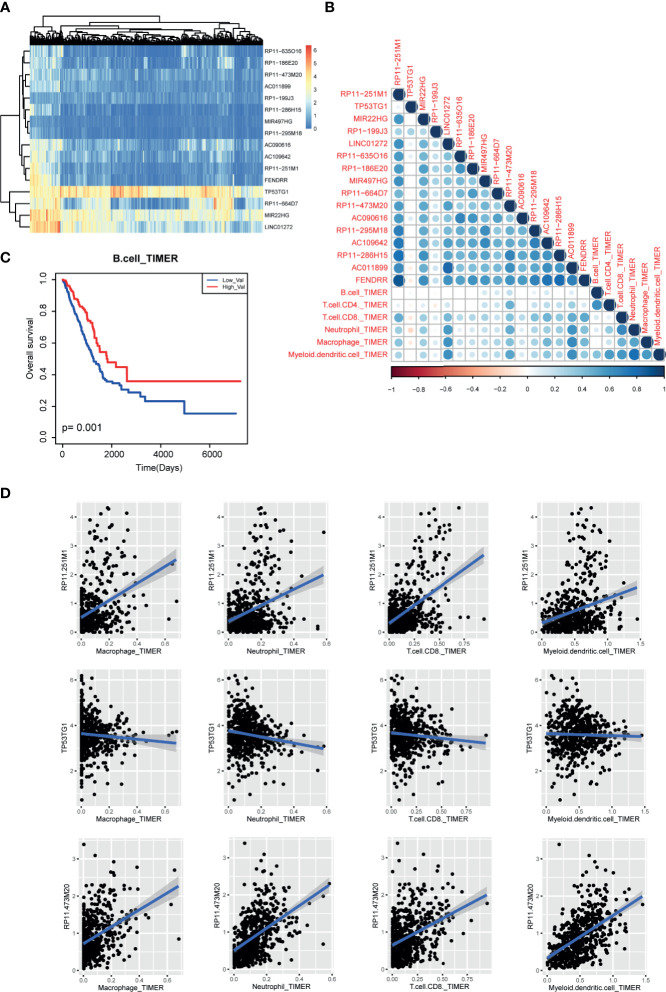
Immune cell infiltration of m6A-related lncRNAs in LUAD patients. **(A)** The expression heatmap of m6A-related lncRNAs in TCGA LUAD cohorts. **(B)** The correlation heatmap of correlation coefficients between m6A regulator expression and TIMER2 immune cell estimation score. **(C)** The Kaplan-Meier survival curves of between B cell-enriched patients and other patients. **(D)** Scatter plots of correlations between m6A regulator expression and TIMER2 immune cell estimation score.

Next, to uncover the potential role of m6A-related lncRNAs in tumor immunology, we collected the pan-can cell infiltration data from TIMER2 database. Based on the above protocol, we selected the lncRNAs with high degree (degree>2) in lncRNA-m6A regulator network as the potential m6A-related lncRNAs. Expression heatmap showed that some lncRNAs showed similar expression patterns in LUAD (**Figure 7A**). Briefly, we calculated the correlation coefficients between m6A-related lncRNAs and immune cell levels by integrating expression data and TIMER2 infiltration data. Results showed that CD8-T cell, neutrophil, macrophage, and myeloid dendritic cell were high related to these lncRNAs (**Figure 7B**). In lncRNA aspect, we found that four lncRNAs were positively related to immune cells than other transcripts, including RP11−251M1, RP11−473M20, LINC01272, and AC011899. And we found that B cell-enriched patients have good prognosis (**Figure 7C**). And lncRNA TP53TG1 showed a reverse tendency (**Figure 7D**). These results also implied that lncRNAs might participate in cancer regulation by controlling immune cell levels in LUAD.

## Discussion

Lung adenocarcinoma is one of the malignant tumors with poor prognosis, which is also a top risk factor of death. However, the molecular mechanism of LUAD is still unknown. Thus, it is urgent to find its molecular mechanism and identify more diagnosis and prognosis biomarkers for LUAD clinical treatments. LncRNA is a type of newfound RNA that is more than 200 bp long and with non-coding capacity. Recent studies have demonstrated that lncRNAs could be used as prognosis markers in LUAD (33, 34). In addition, numerous of lncRNAs have been demonstrated to be involved in the pathological processes of LUAD, such as DANCR, LINC01512, and MALAT1 (35–37). However, there is a lack of global view to comprehensively investigate the lncRNA-related crosstalks. Here, we constructed two types of networks to identify functional lncRNA biomarkers in LUAD, including ceRNA-based TF-lncRNA network and expression correlation-based lncRNA-m6A regulator network. Based on network analysis, some novel lncRNA regulators were identified as potential prognosis markers in LUAD.

Previous studies proposed that TF-lncRNA positive feedback loops have strong prognostic effects in multiple cancers, such as sarcoma and glioma (38, 39). In the field of lung adenocarcinoma, Huang et al. found that FOXP3/lncRNA LINC00520 positive feedback loops facilitated cell proliferative and migratory abilities in LUAD through interaction with miR-3611. Based on these theories, we constructed the LUAD-related TF-lncRNA feedback loop network by integrating gene expression and motif binding. Results showed that core TF-lncRNA feedback loops have strong prognostic effects, such as ELK4 and BDP1 and their lncRNA neighbors. Furthermore, we also found some crucial factors with important clinical values in the positive feedback loops, such as hub TFs CREB1, NFAT5, and POU2F1. CREB1 has been confirmed as the important therapeutic target in lung adenocarcinoma. Jin et al. found the inhibitory role of RBM10 on cell proliferation of lung adenocarcinoma *via* RAP1/AKT/CREB signaling pathway (40). Wang et al. demonstrated that CREB could stimulate GPX4 transcription and inhibit ferroptosis in lung adenocarcinoma (41). Linnerth et al. found that CREB and its associated proteins function in lung adenocarcinoma, and IGF-II induce CREB phosphorylation *via* the Erk5 signaling pathway (42). NFTA5 and POU2F1 were also demonstrated to participate in cancer regulation (43, 44). These results indicated the clinical potentials of these genes in our results. More importantly, we found that the binding sites of TFs on lncRNAs of these TF-lncRNA pairs occurred in both promoter regions and enhancer regions, suggesting the strong regulatory effects between TFs and lncRNAs. Because of the characteristic positive feedback loops, these robust TF-lncRNA pairs could be used for therapeutic targets.

Furthermore, some studies found that m6A modifications of lncRNAs have big impacts on tumor progression (26). Here, we collected 21 m6A regulators to identify lncRNA-m6A regulator crosstalks by expression correlation. As a result, we found that some m6A regulators were good prognostic markers of LUAD, such as RBM15, HNRNPC, and HNRNPA2B1. LncRNA-m6A regulator pairs also exhibited good prognostic ability in LUAD. Moreover, we also investigated the relationships between m6A-related lncRNAs and immune cell infiltration. Results showed that a small subset of lncRNAs was high related to the immune cell purities. For example, LINC01272 was high related to the contents of neutrophil, macrophage, and myeloid dendritic cell, implying the potential immunotherapy for LUAD clinically. We also found that 52 lncRNAs were considered as potential targets in both ceRNA-based network and m6A-based network (**Figure S4**), which indicated that these lncRNAs had important research value in the treatment of LUAD.

In summary, in this study, we constructed LUAD-related lncRNA-TF network and lncRNA-m6A regulator network to identify functional lncRNAs based on gene expression. Firstly, in lncRNA-TF network, network topological analysis revealed some hub lncRNAs and TFs that might control the phenotype of cancers. Module analysis revealed one close lncRNA-related function module, which also exhibited good prognostic performance in LUAD. Furthermore, through integrating ceRNAs strategy and TF motif binding information, we identified some lncRNA-TF positive feedback loops, which could be used as robust prognostic markers and therapeutic targets. Secondly, in lncRNA-m6A network, based on the network analysis results, some key m6A-regulated lncRNAs were identified. We also investigated the relationships between these lncRNAs and immune cell infiltration. All these results provide a new perspective for LUAD prognosis and clinical treatment.

However, our study still has some flaws. Firstly, this study was not focused on subtypes of lung adenocarcinoma. We will investigate the potential role of lncRNAs in subtypes of LUAD to yield more convincing data. Secondly, we used MCODE algorithm to find network modules, which depended on network density. Some modules that were located in the border of network were ignored. Thirdly, in this study, we conducted an integrative bioinformatics analysis to identify the lncRNA-related crosstalks in lung adenocarcinoma, and results indicated that some genes (TFs or lncRNAs) might play vital roles in the subtype cancers. This result also encouraged us to validate the biological function and mechanism. This result also encouraged us to validate the biological function and mechanism of lncRNA-TF mediated regulatory axes. In further studies, we will perform biological experiments to validate and investigate the regulatory mechanism of these factors.

## Data Availability Statement

The original contributions presented in the study are included in the article/**Supplementary Material**. Further inquiries can be directed to the corresponding author.

## Author Contributions

YZ conceived and designed the project. WZ and CB processed the data and performed bioinformatics analysis. CL and LH wrote the manuscript. All authors contributed to the article and approved the submitted version.

## Funding

National Natural Science Foundation of Beijing (No. 7202115).

## Conflict of Interest

The authors declare that the research was conducted in the absence of any commercial or financial relationships that could be construed as a potential conflict of interest.

## Publisher’s Note

All claims expressed in this article are solely those of the authors and do not necessarily represent those of their affiliated organizations, or those of the publisher, the editors and the reviewers. Any product that may be evaluated in this article, or claim that may be made by its manufacturer, is not guaranteed or endorsed by the publisher.

## Supplementary Material

Supplementary Figure S1 Visualization of WGCNA-based lncRNA-TF network.

Supplementary Figure S2 Expression changes of hub genes in **Figure 2** network.

Supplementary Figure S3 Visualization of ATM-related mutations in OncoVAR.

Supplementary Figure S4 Intersection of lncRNAs between m6A-related lncRNA-TF network and ceRNA-based lncRNA-TF network.

Supplementary Table S1 The raw expression data of TCGA LUAD dataset.

Supplementary Table S2 Annotation of hub TFs in OncoVar LUAD-related driver genes.
