# Supplementary figures and images for: Construction and Characterization of Long Non-Coding RNA-Associated Networks to Reveal Potential Prognostic Biomarkers in Human Lung Adenocarcinoma
Source: Front Oncol. 2021 Aug 27;11:720400. doi: 10.3389/fonc.2021.720400 (PMC8430225; doi:10.3389/fonc.2021.720400)

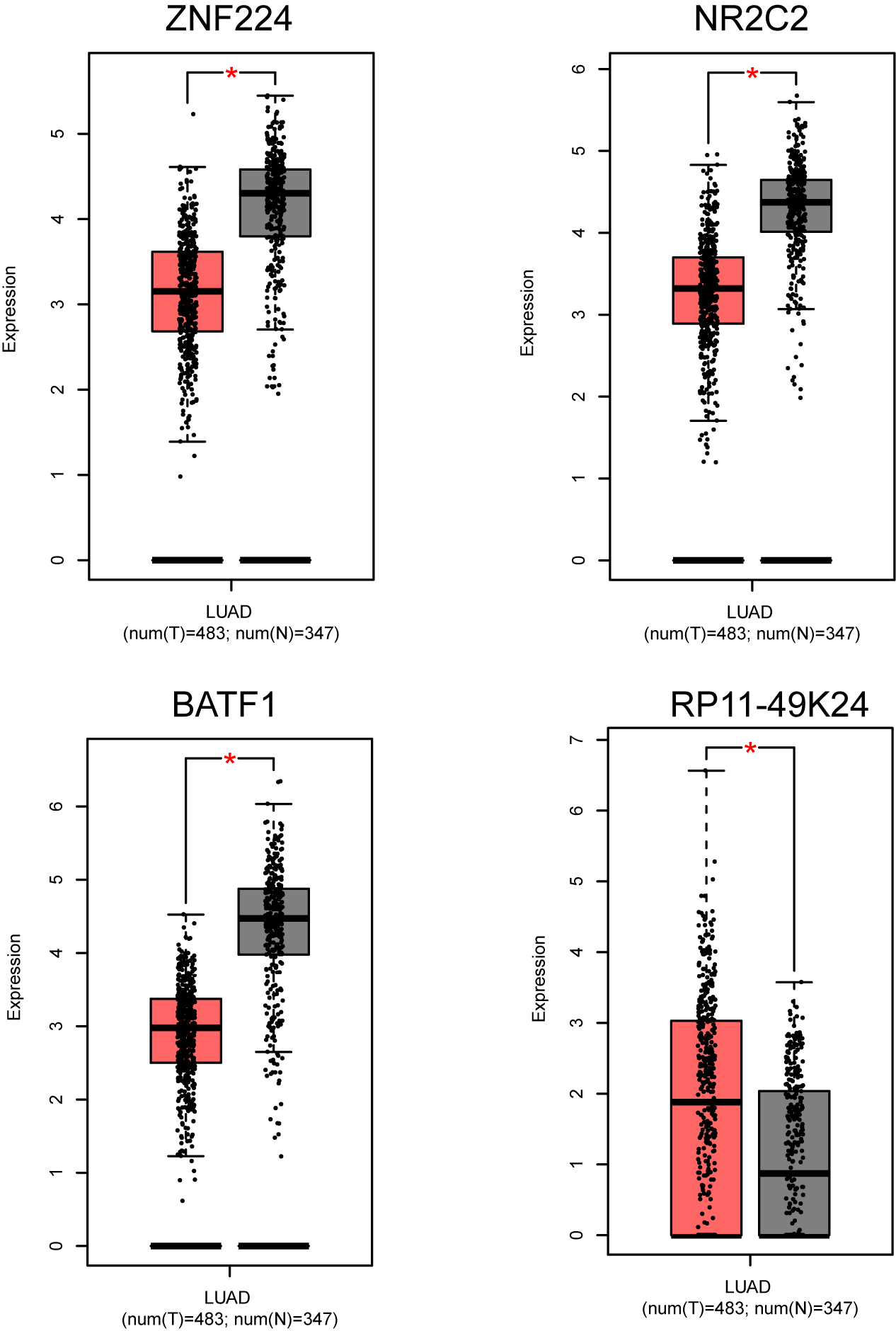

Supplement: Supplementary Figure S1 — Visualization of WGCNA-based lncRNA-TF network. [file Image_1.tif]

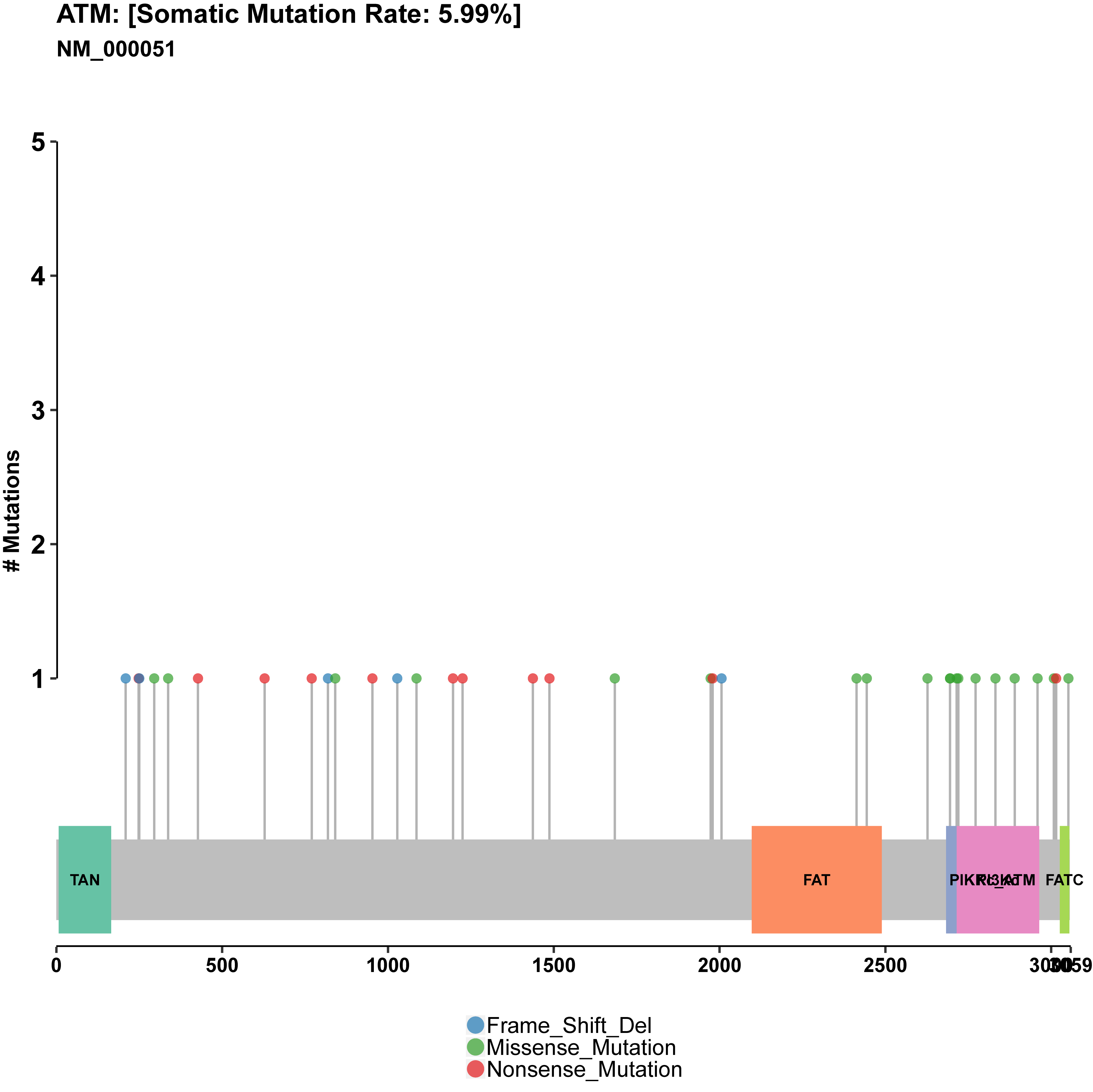

Supplement: Supplementary Figure S2 — Expression changes of hub genes in Figure 2 network. [file Image_2.tif]

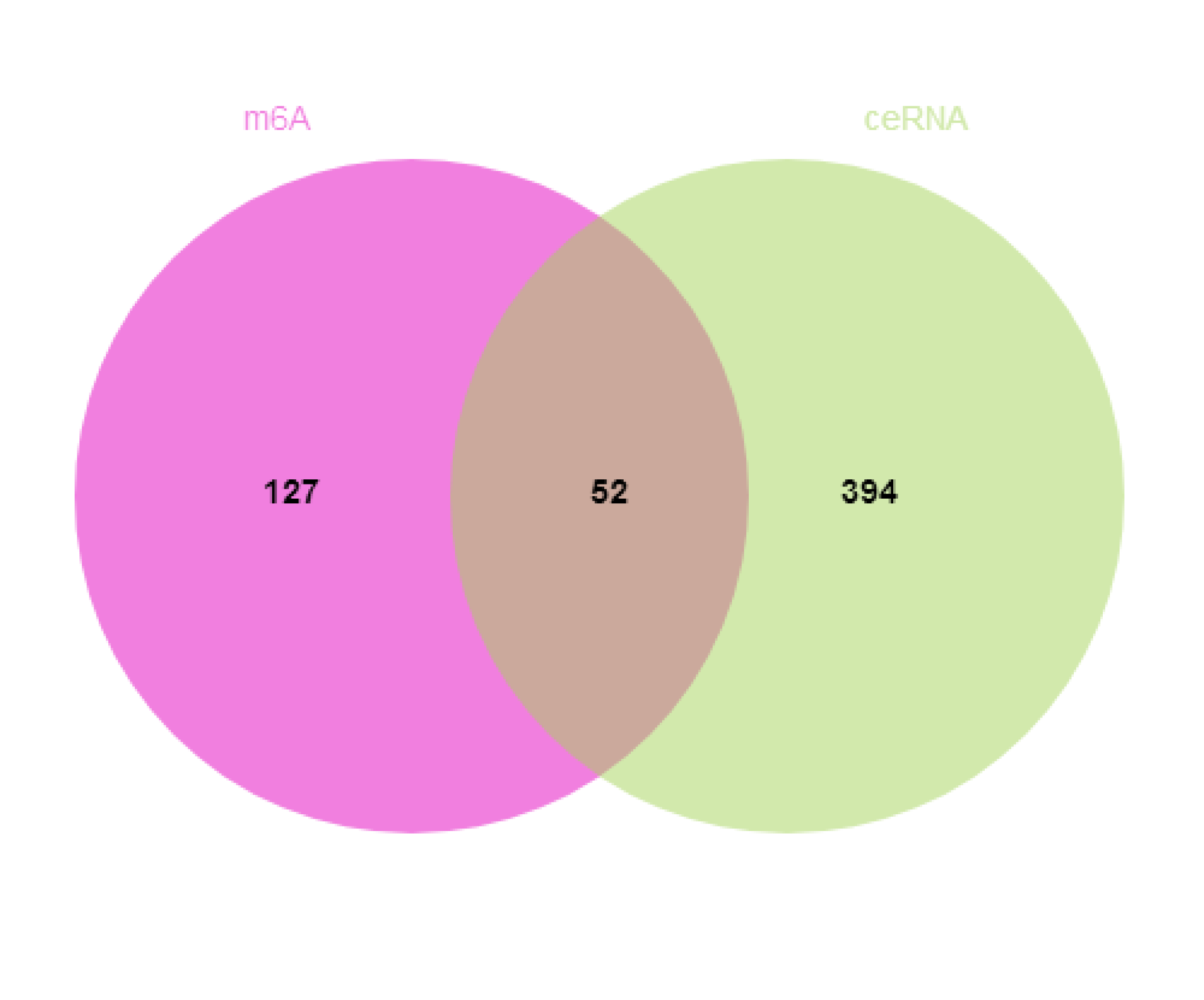

Supplement: Supplementary Figure S3 — Visualization of ATM-related mutations in OncoVAR. [file Image_3.tif]

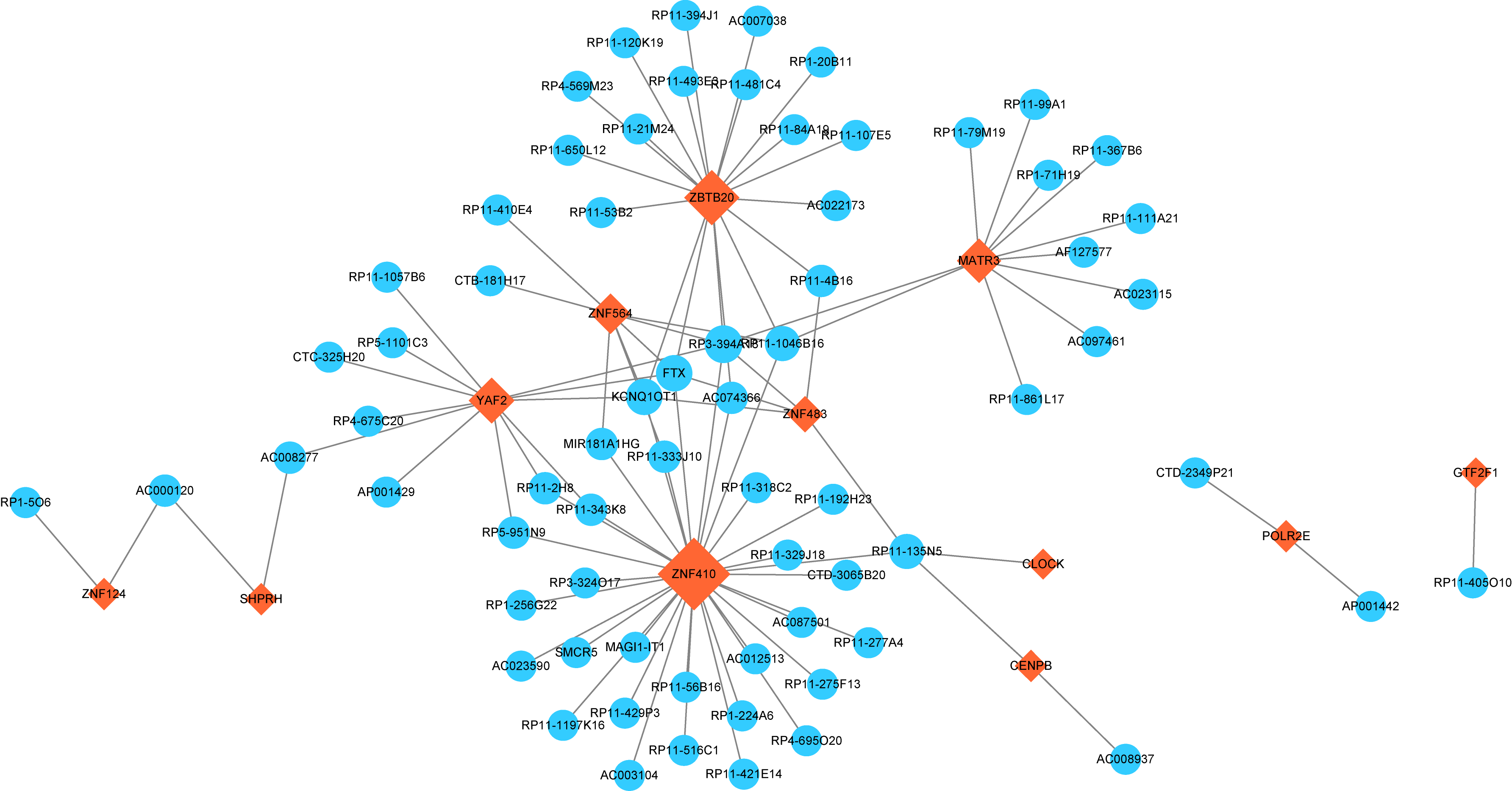

Supplement: Supplementary Figure S4 — Intersection of lncRNAs between m6A-related lncRNA-TF network and ceRNA-based lncRNA-TF network. [file Image_4.tif]
